# BLIMP‐1 and CD39 expression define human effector memory regulatory T cells in blood and recall responses to viruses

**DOI:** 10.1111/imcb.70148

**Published:** 2026-07-14

**Authors:** Chansavath Phetsouphanh, Laura Cook, Katherine J. L. Jackson, Deirdre Cuff, Vera Klemm, Bikash Manandhar, Denise C. Hsu, Yin Xu, John J. Zaunders, Anthony D. Kelleher, Nabila Seddiki

**Affiliations:** ^1^ The Kirby Institute University of New South Wales Sydney New South Wales Australia; ^2^ Department of Microbiology and Immunology University of Melbourne at the Peter Doherty Institute for Infection and Immunity Melbourne Victoria Australia; ^3^ Garvan Institute of Medical Research Darlinghurst New South Wales Australia; ^4^ St. Vincent's Hospital Darlinghurst New South Wales Australia; ^5^ Universite Paris‐Saclay Fontenay‐aux‐roses France; ^6^ Inserm, UMRS1184 Fontenay‐aux‐roses France; ^7^ Immune Diseases, Microbiology and Innovative Therapies (IDMIT), CEA Fontenay‐Aux‐Roses France

**Keywords:** AIM assay, antigen‐specific T cell, CD4^+^ T cell, COVID‐19, IL‐10, Treg

## Abstract

BLIMP‐1, a transcriptional repressor initially identified in plasma B‐cell differentiation, also regulates CD8^+^ T cells and is differentially expressed between naïve and effector/memory CD4^+^ T cells. However, the functions of BLIMP‐1 in human CD4^+^ regulatory T cells (Tregs) remain unclear. We found BLIMP‐1 mRNA levels were low in naïve Tregs from thymus and cord blood, but high in effector/memory Tregs from adult peripheral blood. We confirmed the expression of BLIMP‐1 mRNA by microarray of healthy T‐cell populations and single‐cell RNA‐sequencing of SARS‐CoV‐2 spike‐specific CD4^+^ T cells from convalescent COVID‐19 patients. We found activated memory Tregs expressed the highest levels of BLIMP‐1, FOXP3, and CD39 transcripts and protein. We show that CD39^+^ Tregs, regardless of in vitro or in vivo activation, had the highest levels of BLIMP‐1 protein expression and significantly more IL‐10 mRNA than CD39^neg^ cells. The same result was found through reanalysis of public single cell RNA‐sequencing data from lung (tissue and lymph node) SARS‐CoV‐2 spike‐specific Tregs, where cells with high levels of BLIMP‐1 mRNA had significantly more IL‐10 mRNA than cells with low BLIMP‐1 mRNA. Single‐cell RNA‐sequencing identified an extended transcriptomic profile of these CD39^+^ Tregs that includes the genes encoding HPGD, CTLA‐4, HELIOS, TIGIT, CXCR6, CCR8, and GARP. Together, these data indicate that both circulating and lung resident effector memory Tregs maintain a conserved phenotype following stimulation by cognate antigen. Our data provide further insight into the functions and potential therapeutic targets of the large regulatory T‐cell component of CD4^+^ T‐cell responses to pathogens.

## Graphical abstract

This study found that BLIMP‐1 is highly expressed in activated effector/memory human regulatory T cells, especially CD39^+^ Tregs, where it is associated with increased IL‐10 production and a conserved phenotype across blood and lung tissues during immune responses such as SARS‐CoV‐2 infection.

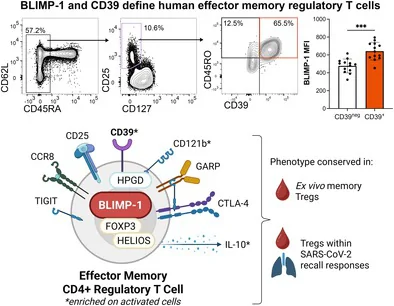

## 1 Introduction

B‐lymphocyte induced maturation protein‐1 (BLIMP‐1) is a zinc finger‐containing transcriptional repressor that is vital for the generation of antibody‐producing plasma cells and an important regulator of effector CD8^+^ T‐cell proliferation, function, and conversion into memory cells [1]. In CD4^+^ T cells, BLIMP‐1 expression is induced by the cytokine IL‐2, but BLIMP‐1 can also repress *IL2* gene transcription via a negative feedback mechanism [2]. Blimp‐1 deficiency in knockout mice results in the accumulation of T cells with activated and memory phenotypes [3, 4]. Interestingly, these studies also showed that Blimp‐1 is expressed in a subset of CD4^+^CD25^+^ regulatory T cells (Tregs) and that Blimp‐1‐deficient cells had impaired Il‐10 production. Tregs derived from the Blimp‐1 knockout mice had preserved in vitro suppression but impaired function in vivo [3, 4].

Many Treg populations have been identified and well‐characterized, although their functional roles are yet to be fully understood [5]. CD4^+^ Tregs that become lineage committed in the thymus (tTregs) are primarily engaged in peripheral control of self‐reactive T cells. Commitment to a Treg lineage can also occur in the periphery (pTregs), following recognition of self or foreign antigen. Tregs are defined by their expression of high levels of IL‐2Rα (CD25) and low levels of IL‐7Rα (CD127) [6], but do not produce IL‐2, requiring exogenous IL‐2 for homeostatic proliferation and function. An important Treg subset is type 1 regulatory T cells (Tr1 cells) that produce high levels of IL‐10, transforming growth factor beta (TGF‐β) and granzyme B (GZMB) and can suppress both adaptive and innate immune responses [7]. BLIMP‐1 appears to be a key regulator of Treg functions, yet its expression and function within human Tregs that have been activated by cognate antigen has not been explored. Here, we investigated whether phenotypically and functionally equivalent BLIMP‐1^+^ Tregs exist in humans and, using ex vivo analysis and in vitro antigen stimulation, characterized single cells at molecular and cellular levels in healthy donors and those living with HIV‐1 or infected by, or vaccinated to, SARS‐CoV‐2.

## 2 Results

### 2.1 BLIMP‐1 Is Highly Expressed in Human Effector/Memory Tregs

**FIGURE 1:**
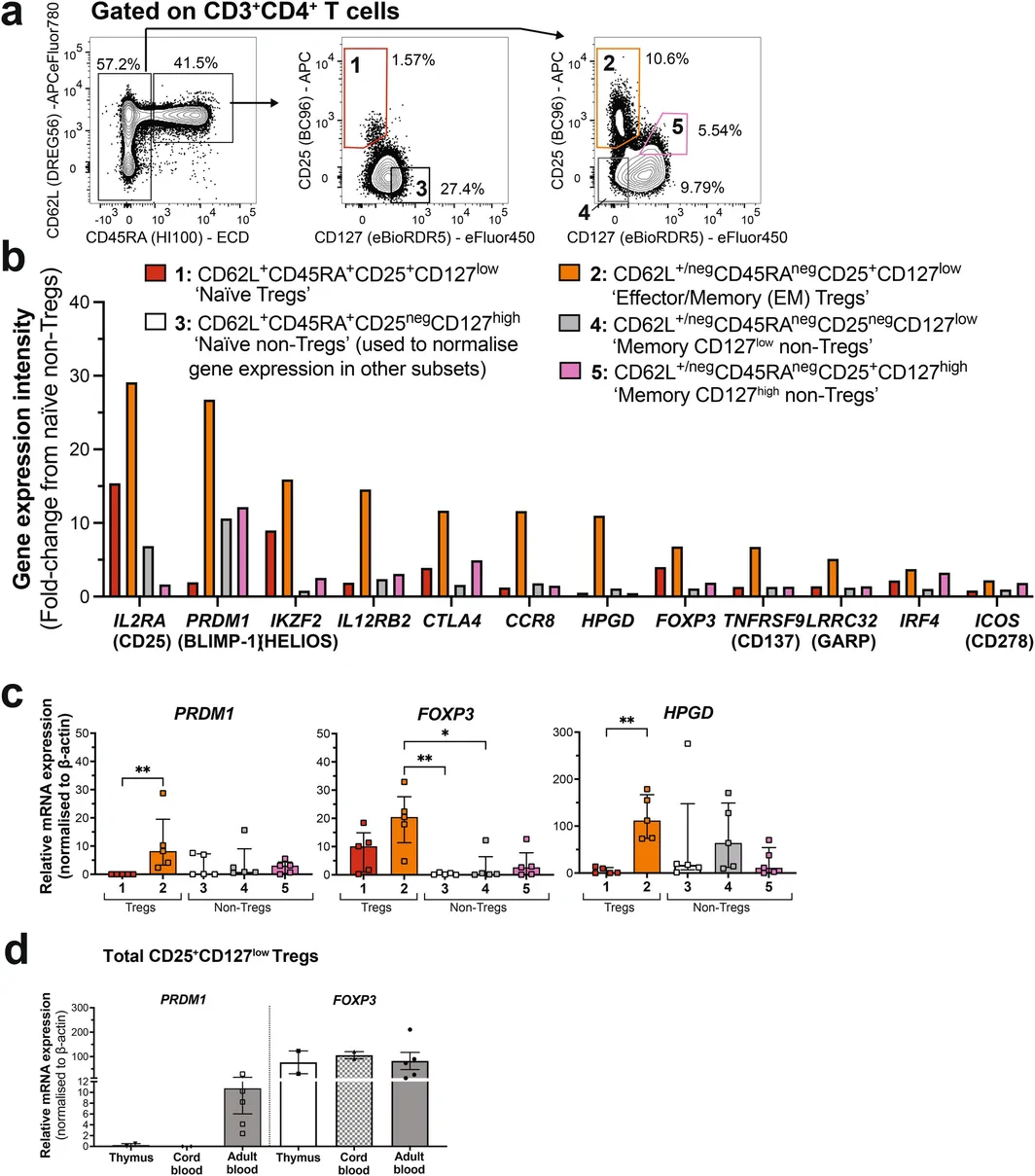
BLIMP‐1 is highly expressed in effector/memory Tregs. (a) Gating strategy for cell sorting CD4^+^ T cell subsets: 1. naïve Tregs (CD62L^+^ CD45RA^+^ CD25^+^CD127^low^), 2. effector/memory Tregs (CD62L^+/neg^CD45RA^neg^CD25^+^CD127^low^), 3. naïve non‐Tregs (CD62L^+^CD45RA^+^CD25^neg^CD127^high^), 4. memory CD127^low^ non‐Tregs (CD62L^+/neg^ CD45RA^neg^CD25^neg^CD127^low^) and 5. memory CD127^high^ non‐Tregs (CD62L^+/neg^CD45RA^neg^CD25^neg^ CD127^high^). (b) Microarray gene expression data from the five cell subsets sorted in (a) for one healthy donor. (c) Ex vivo expression levels of *PRDM1*, *FOXP3*, and *HPGD* mRNA measured by RT‐qPCR for all cell subsets isolated as shown in (a) for *n* = 5 healthy donors. (d) Levels of *PRDM1* and *FOXP3* mRNA detected by RT‐qPCR in total CD25^+^CD127^low^ Tregs isolated from healthy pediatric thymus (*n* = 2), cord blood (*n* = 2), and peripheral adult blood (*n* = 5). Statistical analysis used Kruskal‐Wallis test with Dunn's post‐test for multiple comparisons.

To determine which ex vivo human CD4^+^ T‐cell subset had the highest level of BLIMP‐1 mRNA expression and what other genes were similarly highly expressed, we sorted PBMCs from one healthy individual into 5 subsets of CD4^+^ T cells. These were naïve Tregs (CD62L^+^CD45RA^+^CD25^+^CD127^low^), effector/memory Tregs (CD62L^+/neg^CD45RA^neg^CD25^+^ CD127^low^), naïve non‐Tregs (CD62L^+^CD45RA^+^CD25^neg^CD127^high^), memory CD127^low^ non‐Tregs (CD62L^+/neg^CD45RA^neg^CD25^neg^CD127^low^), and memory CD127^high^ non‐Tregs (CD62L^+/neg^CD45RA^neg^ CD25^neg^ CD127^high^) (Figure 1a). RNA was extracted from each subset, and micro‐array analysis was performed using the Affymetrix microarray platform. We found that *PRDM1*, the gene encoding BLIMP‐1, had the highest expression in the effector/memory (EM) Treg compartment, being 14‐fold greater than in naïve Tregs and 27‐fold higher than naïve CD4^+^ non‐Treg cells (Figure 1b). Compared to all other subsets, the other most highly differentially expressed genes in EM Tregs were *IL2RA* (CD25), *IKZF2* (HELIOS), *IL12RB2*, *CTLA4, CCR8, HPGD, FOXP3, TNFRSF9* (CD137), and *LRRC32* (GARP). Memory CD127^low^ and CD127^high^ non‐Treg cells expressed moderate levels of *PRDM1*, but this was less than half the level in EM Tregs. Gene expression levels for *PRDM1, FOXP3*, and *HPGD* were validated by RT‐PCR in *n* = 5 adult healthy controls as being highest within memory Tregs (Figure 1c). *PRDM1* expression was low in total Tregs isolated from thymus (*n* = 2) and cord blood (*n* = 2), which were predominantly naïve cells, but significantly higher in Tregs from adult peripheral blood that are ~50% memory (CD45RO^+^) [8]. In contrast, the “master” transcription factor of Tregs, *FOXP3*, was highly expressed in all samples as it is required for thymic lineage commitment (Figure 1d). These data suggest BLIMP‐1 expression in Tregs is developmentally regulated and is only expressed in EM Tregs.

### 2.2 Antigen Stimulated SARS‐CoV‐2‐Specific Tregs Are Enriched for BLIMP‐1 and CD39

**FIGURE 2:**
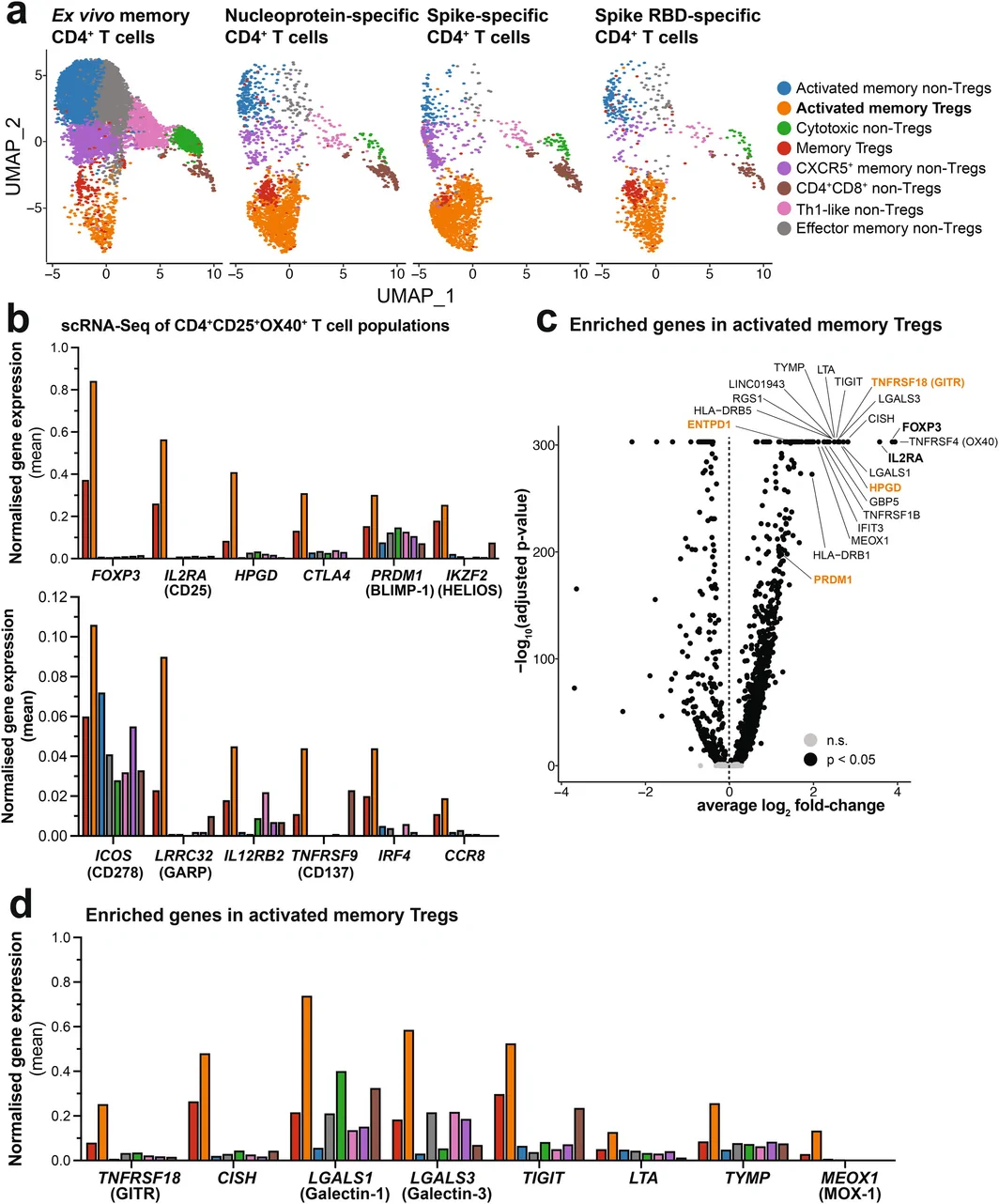
Antigen‐specific memory Tregs express high levels of BLIMP‐1 and CD39. (a) Single‐cell RNA‐seq analysis of ex vivo memory (CD45RO^+^) CD4^+^ T cells and CD4^+^ CD25^+^OX40^+^ T cells sorted following 48 h stimulation with the indicated SARS‐CoV‐2 antigens. Colors discriminate the 8 clusters of cells identified in the dataset, with activated memory Tregs colored orange (data are from *n* = 4 COVID‐19 convalescent individuals). (b) The 8 clusters identified in (a) were analyzed for expression levels of genes identified as highly expressed within ex vivo effector/memory Tregs in Figure 1a. (c) Volcano plot showing differential gene expression analysis for activated memory Tregs compared to all other subsets. The most significantly upregulated genes are in the top right quadrant (black dots: *p* < 0.05). (d) The next top 8 highly expressed genes in the activated memory Treg subset compared to other subsets after excluding the genes shown in (b).

We have previously shown that following 48‐h antigen stimulation, antigen‐specific CD4^+^ T cells can be detected by induced upregulation of CD25 and OX40 (CD134) [9] and that CD39^+^ co‐expression identifies the subset enriched for FOXP3^+^ Tregs [10]. Here, we used single‐cell RNA‐sequencing (scRNA‐seq) to confirm whether antigen‐specific memory CD4^+^ Tregs within an in vitro recall response were enriched for BLIMP‐1 expression. We used PBMCs that had been collected from *n* = 4 SARS‐CoV‐2 convalescent individuals from the ADAPT study [11] and stimulated with SARS‐CoV‐2 (Wuhan strain) nucleoprotein, spike or spike RBD peptide pools for 44‐h. We then sorted the antigen‐specific CD25^+^OX40^+^ CD4^+^. T cells, using unstimulated memory CD4^+^ T cells sorted as the comparator subset. scRNA‐seq was performed on a total of 14 053 CD4^+^ T cells comprising: (i) 9039 ex vivo (unstimulated) memory cells; (ii) 2026 nucleoprotein‐specific cells; (iii) 1989 spike‐specific cells; and (iv) 999 RBD‐specific cells. Eight clusters of CD4^+^ T‐cell subsets were identified, including two Treg subsets annotated as “memory” and “activated memory” Tregs (Figure 2a). We first assessed levels of genes found to be highly expressed in EM Tregs in our microarray data (Figure 1b). Similarly, we found that ‘activated memory’ Tregs had the highest expression of *PRDM1* (BLIMP‐1) along with *FOXP3, IL2RA* (CD25), *HPGD, CTLA4, IKZF2* (HELIOS), *ICOS, LRRC32* (GARP), *IL12RB2, TNFRSF9* (CD137), *IRF4*, and *CCR8* compared to other CD4^+^ T‐cell subsets (Figure 2b). Therefore, the gene profile of total ex vivo memory Tregs is very similar to that in antigen‐specific, stimulated Tregs. Unbiased differentially expressed gene analysis of SARS‐CoV‐2‐specific “activated memory” Tregs also confirmed that *ENTPD1* (CD39) was one of the highest expressed genes compared to all other subsets, supporting that CD39^+^ Tregs, which are enriched within antigen recall CD4^+^ T‐cell responses [10], have the highest expression of *PRDM1* (BLIMP‐1) (Figure 2c). scRNA‐seq revealed additional genes highly expressed in “activated memory” SARS‐CoV‐2 specific Tregs, including *TNFRSF18* (GITR), *CISH, LGALS1* (Galectin‐1), *LGALS3* (Galectin‐3), *TIGIT, LTA, TYMP*, and *MEOX1* (MOX‐1) (Figure 2d). Of these, GITR and TIGIT are well‐known Treg‐associated markers [12, 13], with a previous study finding a role for galectin‐1 in mediating Treg suppression in mice and humans [14] likely through binding glycans on the surface of target cells [15].

### 2.3 BLIMP‐1 Is Highly Expressed Within CD39 ^+^ Tregs and Associates With IL‐10 Secretion

**FIGURE 3:**
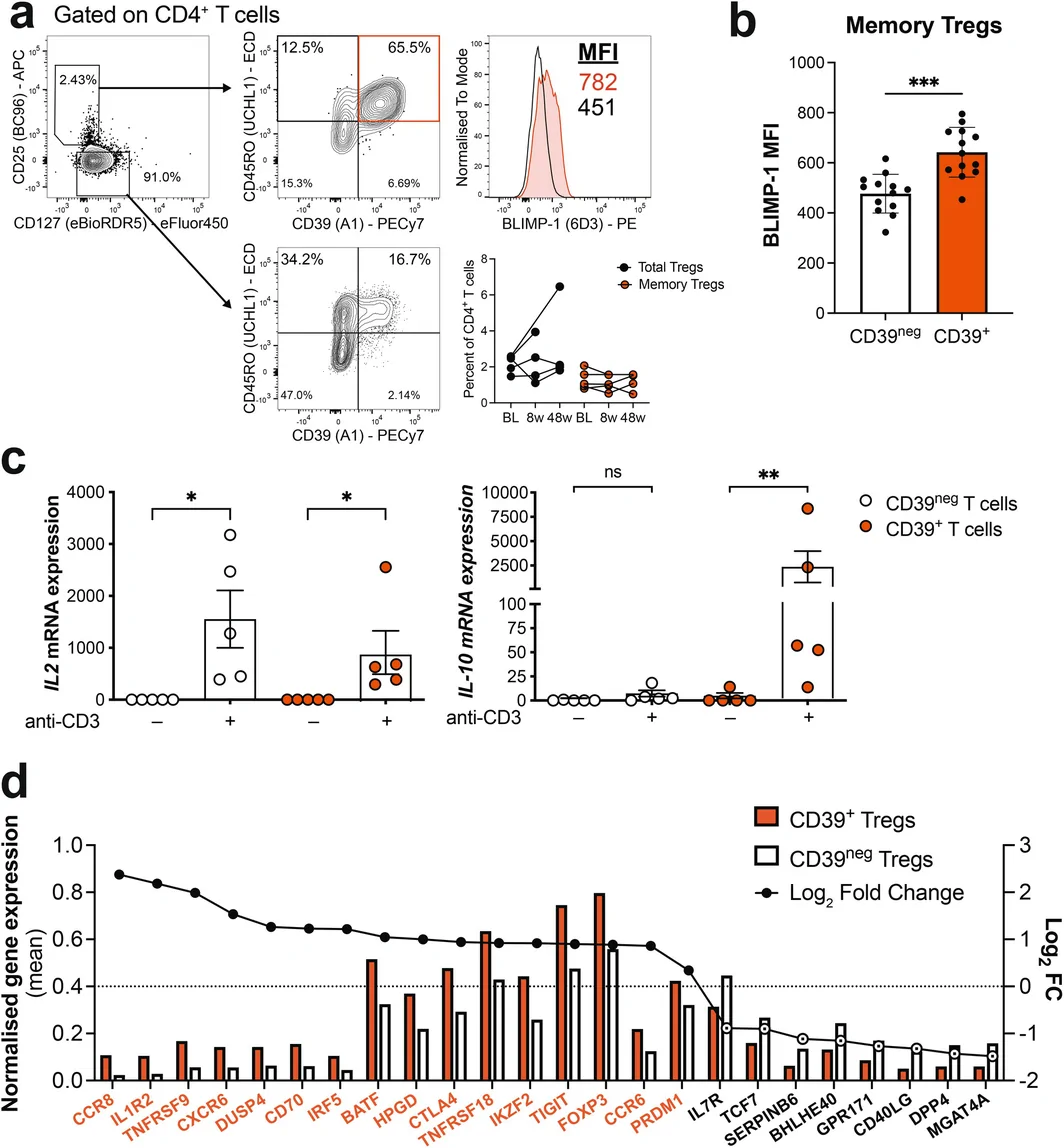
BLIMP‐1 expression is highest within CD39^+^ cells and is associated with IL‐10 production. (a) As part of a clinical trial of treating HIV‐infected patients with IL‐2, we measured longitudinal Treg percentages and phenotype in intravenous IL‐2 treatment arm (*n* = 5) at baseline pre‐treatment (BL) and at 8 weeks (w) and 48w post treatment (*n* = 4–5 individuals per time point). (b) BLIMP‐1 expression levels are shown within memory (CD45RO^+^) CD39^+^ and CD39^neg^ Tregs from samples at all time points (*n* = 13). Analysis used Wilcoxon matched pairs signed rank test. (c) CD4^+^CD45RO^+^CD39^+^ or CD39^neg^ T cells were sorted from *n* = 5 healthy PBMCs and stimulated for 24‐h with 1 μg/mL anti‐CD3 then RT‐qPCR was used to detect *IL‐2* and *IL‐10* mRNA levels (Mann–Whitney U‐tests). (d) The activated memory Treg cluster from the single cell RNA‐seq dataset shown in Figure 2 (all antigen‐stimulated conditions combined) was divided into CD39^+^ and CD39^neg^ cells. The differentially expressed genes are shown between these subsets, with bars showing normalized mean gene expression and dots depicting log_2_ fold change. Genes are listed left to right from highest differentially expressed in CD39^+^ Tregs (black dots) to those more highly expressed in CD39^neg^ Tregs (white dots).

We have previously shown that chronic HIV‐infected patients have increased BLIMP‐1 expression in CD4^+^ T cells, which is further increased upon IL‐2 and TCR stimulation in vitro [16]. Here, we investigated whether IL‐2 therapy modulated patterns of BLIMP‐1 expression in Tregs in vivo. We analyzed blood from chronic, treated HIV‐infected individuals who were randomized to the intravenously infused IL‐2 treatment arm of a phase 2 clinical trial of IL‐2 therapy, given daily for 5 days every 8 weeks over 5 cycles [17]. Samples were examined longitudinally at 3 time points: baseline (BL); 8 weeks post therapy commencement (after Cycle 1); and 48 weeks post therapy commencement, being 8 weeks after Cycle 5. We assessed the proportions of circulating total and memory Tregs and the co‐expression of BLIMP‐1 and CD39 protein (Figure 3a). We did not detect significant expansion of any Treg population (Figure 3a). However, regardless of IL‐2 therapy duration, memory CD39^+^ Tregs had significantly higher expression of BLIMP‐1 compared to CD39^neg^ Tregs (Figure 3b). Therefore, CD39 and BLIMP‐1 co‐expression remains consistent in healthy individuals, both ex vivo and after in vitro activation, and in HIV‐infected patients, before and after in vivo IL‐2 treatment.

There is already a well‐established link between BLIMP‐1 and IL‐10 in mice and humans [1], so we investigated whether the CD39^+^ cells, with highest BLIMP‐1 expression, were also enriched for IL‐10 production. As *IL‐10* transcript levels were undetectable in our ADAPT study scRNA‐seq dataset, we sorted total CD4^+^ T cells into CD39^+^ and CD39^neg^ subsets and stimulated with anti‐CD3 for 24 h. Total CD4^+^ T cells, rather than Tregs, were used to provide sufficient cells for mRNA extraction. Using RT‐qPCR we found elevated *IL‐2* in both subsets after activation, but only CD39^+^ T cells had increased levels of *IL‐10* mRNA (Figure 3c).

The surface marker CD39^+^ is useful for isolating populations of viable antigen‐specific FOXP3^+^ Tregs that are enriched for BLIMP‐1 and IL‐10. Therefore, we analyzed our SARS‐CoV‐2‐specific scRNA‐seq dataset to identify an extended gene signature of these cells (Figure 3d). As expected, the genes with the highest normalized mean expression in CD39^+^ Tregs were *FOXP3, PRDM1* (BLIMP‐1), *CTLA4, IKZF2* (HELIOS), and *TIGIT*. Additional highly differentially expressed genes in the CD39^+^ Tregs were *CCR8, IL1R2* (CD121b), *CXCR6, DUSP4*, *CD70, IRF5, BATF, TNFRSF18* (GITR), and *CCR6*. Although protein expression validation is still required, those with confirmed cell surface expression may be useful in combination with CD39 to isolate activated memory BLIMP‐1^+^ Tregs.

We also analyzed IL‐10 protein expression/secretion by flow cytometry within activated CD4^+^ T cells to assess co‐expression with BLIMP‐1 and CD39. IL‐10 staining for flow cytometry is notoriously challenging, as it is highly sensitive to activation conditions, feedback mechanisms and protocol variations [18]. There were too few antigen‐specific IL‐10^+^ cells to combine IL‐10 detection with a CD25/OX40 assay using peptide antigen. We therefore used two complementary approaches and obtained one dataset per approach (*n* = 2). We first expanded PBMCs from a CMV‐seropositive donor for 8 days with CMV lysate followed by 6‐h stimulation with PMA and ionomycin in the presence of Brefeldin A. As cells were highly activated and proliferating, neither the CD25^+^CD127^low^FOXP3^+^ nor CD25^+^OX40^+^CD39^+^FOXP3^+^ definitions of Tregs could be used without significant contamination of non‐Tregs; instead Tregs were identified as FOXP3^+^HELIOS^+^ cells [19]. Compared with FOXP3^neg^HELIOS^neg^ non‐Tregs, FOXP3^+^HELIOS^+^ Tregs expressed higher levels of CD39, BLIMP‐1, and IL‐10 as measured by MFI (Figure S2a). This result was corroborated using a 48‐h stimulation with CytoStim, a positive control reagent that crosslinks MHC and TCR. This induced CD25 and OX40 expression on the majority of cells, which were subsequently assessed for IL‐10 secretion using a cytokine capture assay following an additional 1‐h anti‐CD3/CD28/CD2 stimulation. The IL‐10^+^ cells exhibited increased BLIMP‐1 MFI compared with IL‐10^neg^ cells (Figure S2b). While further experiments and staining optimization are required to confirm BLIMP‐1 and IL‐10 protein co‐expression, these data are consistent with our transcriptomic findings.

### 2.4 BLIMP‐1 Defines a Conserved Effector Treg State in Tissue

**FIGURE 4:**
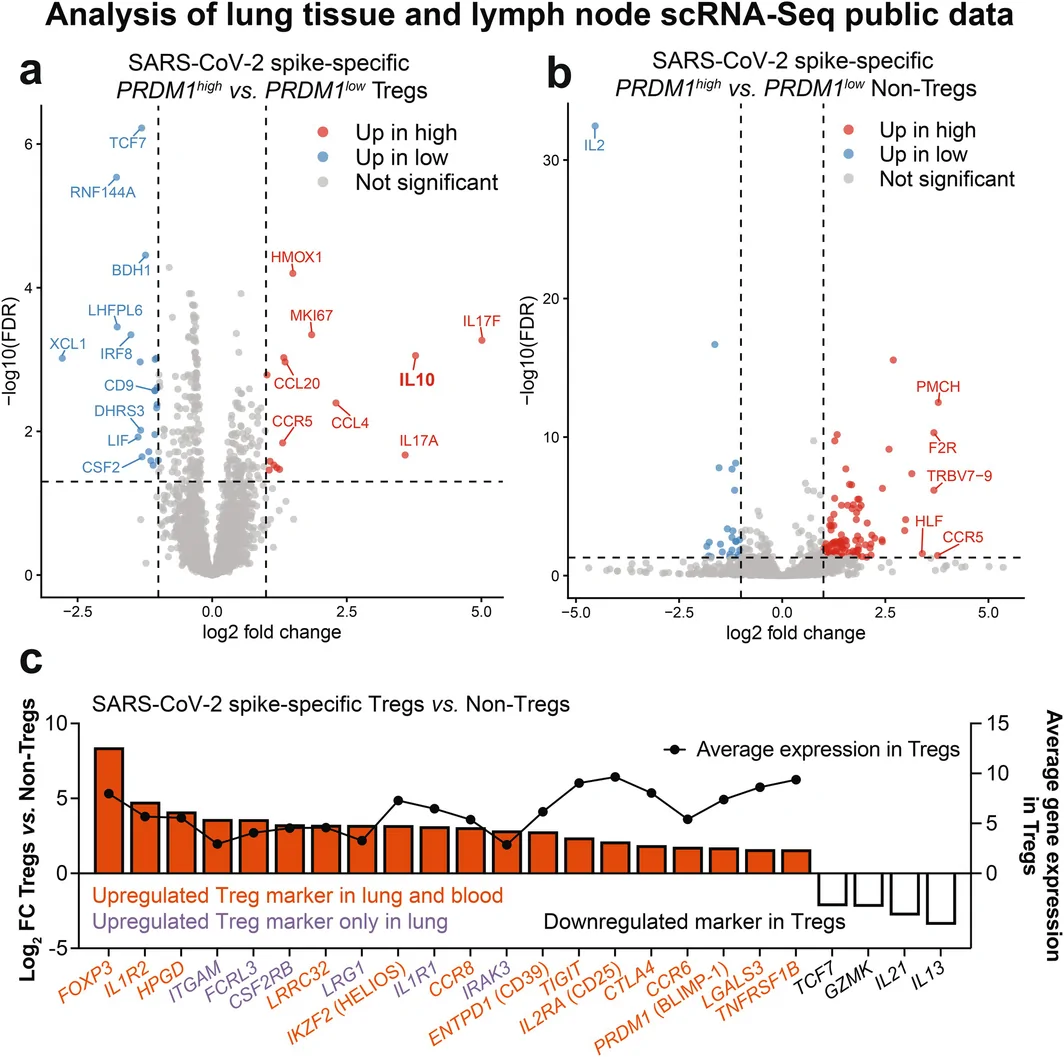
Transcriptional profiles of SARS‐CoV‐2 spike specific lung Treg populations. We have analyzed publicly available data from Davis‐Porada et al. [20]. Briefly, the authors isolated mononuclear from lung tissue and lung lymph nodes of *n* = 6 human organ donors. Cells were stimulated with a SARS‐CoV‐2 spike peptide pool or unstimulated for 24‐h in vitro. Spike‐specific CD4^+^ T cells were identified by co‐expression of surface markers OX40 and 4‐1BB and sorted for single cell sequencing. Read count matrices were obtained under accession GSE261278 using the authors CD4^+^ T cell subset annotations. We stratified the (a) Tregs and (b) non‐Tregs by *PRDM1* expression based on normalized RNA expression levels (PRDM1^low^ < 0.32 and PRDM1^high^ > 0.70, see Supplemental Figure 2c). The volcano plots show results from differential gene expression analysis that included donor, tissue, and stimulation condition as covariates. Horizontal and vertical dashed lines indicate FDR = 0.05 and log_2_FC = 1 thresholds, respectively. (c) We compared the differentially expressed genes in the total spik‐specific Tregs compared to non‐Tregs in the lung samples and represent the log_2_ FC for significant genes along with average (mean) expression in the Tregs, identified conserved genes with our analysis of blood responses.

Our scRNA‐seq data established that BLIMP‐1 is highly enriched within antigen‐specific “activated memory” Tregs in peripheral blood; we next asked whether this transcriptional program is preserved at sites of immune activation in tissue. To address this, we analyzed a publicly available scRNA‐seq dataset from human lung tissue and lung draining lymph nodes generated by Davis‐Porada et al. [20], where we stratified SARS‐CoV‐2 spike‐specific CD4^+^ T cells by *PRDM1* (BLIMP‐1) expression (cutoffs defined in Figure S2c). Importantly, we found the lung tissue/node‐derived SARS‐CoV‐2‐specific Tregs that were *PRDM1* ^*high*^ had significantly higher expression of *IL10* than *PRDM1* ^*low*^ Tregs. The *PRDM1* ^*high*^ cells additionally had increased levels of *IL17F, IL17A, CCR5, CCL20, MKI67* (Ki‐67), and *HMOX1*, but not *ENTPD1* (CD39) transcripts (Figure 4a). However, we found *PRDM1* transcripts were slightly, yet significantly, enriched in *ENTPD1* ^*high*^ vs. *ENTPD1* ^*low*^ Tregs (0.6 log_2_ fold change, *p* < 0.0001, data not shown). In contrast, SARS‐CoV‐2 spike‐specific *PRDM1* ^*low*^ tissue Tregs preferentially expressed genes associated with less differentiated, or naïve, states, such as *TCF7* and *RNF144A*, indicating that BLIMP‐1 (PRDM1) marks functional specialization of Tregs within inflamed tissues and within immune responses to viral antigens.

This BLIMP‐1‐associated signature was largely restricted to the Treg lineage, as *PRDM1* ^*high*^ versus *PRDM1* ^*low*^ SARS‐CoV‐2 spike‐specific non‐Tregs from the same dataset had a more heterogeneous transcriptional profile and *IL10* levels were not enriched, indicating the BLIMP‐1 and IL‐10 association is a feature of Tregs (Figure 4b). Finally, similar to our analysis of blood Tregs (Figure 3d), we analyzed the extended suite of enriched markers within the lung SARS‐CoV‐2 spike‐specific Tregs compared to the non‐Tregs. We found many of these markers were conserved with our blood signature including *PRDM1* and *ENTPD1* (CD39) along with core regulatory genes *FOXP3, IL2RA, CTLA4*, and *IKZF2*, and markers including *ITGAM* and *IL1R1* that may be unique to lung Tregs (Figure 4c). Taken together, these data extend our blood‐based findings and demonstrate that BLIMP‐1 defines a conserved population of effector memory Tregs that, regardless of activation, in blood and tissue are enriched for *IL10*.

## 3 Discussion

In this study, we determined that BLIMP‐1 is highly expressed within CD39^+^ effector/memory Tregs from blood, both ex vivo unstimulated and viral antigen‐specific cells. We found BLIMP‐1 is expressed as cells mature, with very low mRNA levels in naïve Tregs from thymus and cord blood, and high expression in adult blood Tregs. This was corroborated by our microarray and scRNA‐seq data, where, compared to all other CD4^+^ T cell subsets, *BLIMP‐1* was highly expressed in effector/memory (EM) Tregs. Human *BLIMP‐1‐*expressing EM Tregs had the highest expression of *FOXP3, IL2RA* (CD25), *HPGD, CTLA4, IKZF2* (HELIOS), *ICOS, LRRC32* (GARP), *IL12RB2, TNFRSF9* (CD137), *IRF4*, and *CCR8* transcripts. This profile was also found, following 48‐h of antigen stimulation, in SARS‐CoV‐2 specific “activated memory” Tregs. Of these core markers, CD25, FOXP3, CTLA‐4, HELIOS, and GARP are all well‐described Treg markers that are associated with suppressive function [5]. A recent study has shown that FOXP3 and HELIOS optimally detects Tregs within antigen‐specific cells characterized by OX40 (CD134) and 4‐1BB (CD137) expression [21]. IRF4 has been linked to BLIMP‐1 expression in mouse Tregs, and along with CCR8, shown to be expressed on suppressive intratumor Tregs in humans [22, 23]. Both IRF4 and ICOS, along with BLIMP‐1 have been proposed as markers of IL‐10‐secreting type 1 regulatory (Tr1) cells [24, 25]. IL‐12RB2 has been associated with functional maturation of murine Tregs [26] and the co‐stimulatory molecule CD137 (4‐1BB, *TNFRSF9*), together with an absence of CD40L (CD154) can discriminate antigen‐specific Tregs from non‐Tregs [27]. Many of these markers, including the enzyme hydroxyprostaglandin dehydrogenase (HPGD) are part of a proposed 31‐gene core signature of ex vivo human Tregs, which notably does not include *PRDM1* [28]. Importantly, we show here that these markers are conserved within activated viral‐specific CD39^+^ Tregs, which are found in activation induced marker assays (CD25/OX40) to comprise a large (usually ≥ 50%) proportion of antigen‐specific CD4^+^ T cells in blood [10], and are even higher in tissues (> 80%) [20]. Intravenous IL‐2 treatment in HIV‐infected patients did not affect this phenotype, with CD39^+^ Tregs having significantly higher BLIMP‐1 protein expression compared to CD39^−^ Tregs. However, in these patients BLIMP‐1 protein was also enriched within CD39^+^ non‐Treg cells, compared to CD39^neg^ cells (data not shown). Therefore, in this setting BLIMP‐1 expression is no longer unique to Tregs but remains coupled to CD39 expression in CD4^+^ T cells.

We used our scRNA‐seq dataset to define an extended transcriptome for activated memory SARS‐CoV‐2 spike specific Tregs. In addition to *PRDM1*, we identified uniquely high expression of *CCR8, IL1R2* (CD121b), *CXCR6, DUSP4*, *CD70, IRF5, BATF, TNFRSF18* (GITR), and *CCR6*. Interestingly, analysis of a public scRNA‐seq dataset [20] revealed these same markers are also enriched in SARS‐CoV‐2 spike‐specific Tregs isolated from lung and lung‐associated lymph nodes, compared to non‐Tregs. Importantly, this analysis also confirmed that lung SARS‐CoV‐2 spike‐specific *PRDM1* ^*high*^ Tregs, but not non‐Tregs, are significantly enriched for *IL10*. These findings show that across both blood and tissue, BLIMP‐1 is a defining feature of activated antigen‐specific FOXP3^+^ Tregs and is closely associated with IL‐10 expression, identifying BLIMP‐1 as a marker of IL‐10 producing Tregs during viral antigen recall responses.

Further work is required to define the precise contribution of BLIMP‐1 in regulating the expression of IL‐10 in CD39^+^ Tregs, a function shown to be important for generating cellular immunity in mice [29]. Of note, mouse studies suggest that BLIMP‐1 is dispensable for Treg in vitro suppressive function [3, 4]; therefore, its contribution to human Treg suppressive function should also be explored. Our scRNA‐seq analysis also indicates that CD39 may not be the best marker to identify lung SARS‐CoV‐2‐specific BLIMP‐1^+^ Tregs, which requires further exploration at the protein level and across more tissues and antigen responses. Additional genes identified in the SARS‐CoV‐2 spike‐specific CD39^+^ Tregs may point to potential homing and other functions of these cells within a viral antigen stimulated response. The IL‐10/BLIMP‐1 axis is a potentially critical homeostatic mechanism that may govern the function of circulating CD39^+^ Tregs. Given its surface accessibility, CD39 may therefore represent a promising immunotherapeutic target on blood Tregs in the context of viral infections.

## 4 Materials and Methods

### 4.1 Study Participants

Healthy donors were recruited through St Vincent's Hospital with ethics approval from St Vincent's Hospital, Sydney Human Research Ethics Committee (HREC/13/SVH/145) or internally at the Doherty Institute with ethics approval from the University of Melbourne Human Research Ethics Committee (21201‐56281). We also used data from *n* = 4 subjects enrolled in ADAPT, an ongoing prospective, observational cohort study of patients seen at St Vincent's Hospital Sydney (Australia) who tested PCR‐positive for SARS‐CoV‐2 infection; this cohort has previously been described in detail [11]. The ADAPT study was approved by St. Vincent's Hospital, Sydney Human Research Ethics Committee (2020/ETH00964) and is a registered trial (ACTRN12620000554965). Normal thymus specimens were from children aged 1–7 months undergoing corrective cardiac surgery at the Children's Hospital at Westmead with approval from the Hospital Ethics Committee. Cord blood mononuclear cells were isolated from healthy infants after uncomplicated births, after parents' approval. The HIV^+^ IL‐2 therapy cohort enrolled HIV^+^ subjects if they had been stable on ART for at least 2 months prior to screening, with a CD4^+^ T cell count between 200 and 500 cells/μL and relatively normal hematological and biochemical profiles. Subjects were excluded if they had a history of previous IL‐2 therapy, prior AIDS‐defining clinical condition other than muco‐cutaneous Kaposi's Sarcoma, or concurrent use of unlicensed anti‐viral agents, corticosteroids, chemotherapeutic agents, or immunomodulatory agents. Once written informed consent was given, subjects were randomized to one of three arms: (A) continued anti‐retroviral therapy (ART) alone, (B) ART with polyethylene glycol as subcutaneous injections, or (C) ART with 12 × 10^6^ IU/day over 5‐day cycles of recombinant IL‐2 given as intravenous infusion. Treatment cycles were every 8 weeks and samples were collected at appropriate time points after up to 5 cycles [17]. The study was approved by St Vincent's Hospital, Sydney Human Research Ethics Committee (HREC/12/SVH/298, article number: SVH 12/217).

### 4.2 Flow Cytometry

Flow cytometry was performed on a 4‐laser LSR‐II (BD Biosciences) or a 5‐laser Aurora cytometer (Cytek Bioscience). Staining procedures were as previously described [10] and used CD3‐PerCPCy5.5, ‐PerCP (Clone: SK7) or ‐RB744 (UCHT1), CD8‐BUV805 (RPA‐T8) or ‐BUV615 (SK1), CD4‐AF700, ‐BUV395 (RPA‐T4) or ‐BB700 (SK3), CD62L‐APCeFluor780 (DREG56), CD25‐APC (BC96) or ‐BB515 (M‐A251), OX40‐BV421 (L106), CD39‐BV421 (A1) or ‐RB780 (Tu66) (BD Biosciences), CD45RA‐ECD (Beckman Coulter) and CD127(eBioRDR5)‐eFluor450 (ThermoFisher Scientific). All surface markers were used at 1:100, except for CD25, OX40 and CD39 that were 1:50. Surface staining panels included 1:1000 fixable viability dye eFluor780 (ThermoFisher Scientific) and 1:25 of Brilliant Stain Buffer Plus (BD Biosciences). Intracellular staining followed fixation and permeabilization with FoxP3 transcription factor buffer set following manufacturers protocol (ThermoFisher scientific). We used 1:100 FOXP3‐RB613 (259D/C7) and 1:50 HELIOS‐RY610 (22F6) (BD Biosciences) and either rat anti‐human BLIMP‐1‐PE (6D3) monoclonal antibody at 1:200 dilution (sc‐47 732; Santa‐Cruz) or BLIMP‐1‐AF647 (6D3) at 1:50 dilution (BD Biosciences). Analyses were performed using FlowJo v10.10.1 (BD Biosciences). Cell sorting was performed on the FACS ARIA‐II and ARIA‐III (BD Biosciences).

### 4.3 CD25/OX40 AIM Assay

Peripheral blood mononuclear cells (PBMC) were isolated from EDTA anti‐coagulated blood within 4‐h of venepuncture. Antigen‐specific CD4 T cells responding to recall antigens, simultaneously up‐regulating CD25 and OX40 (CD134), were measured in cultures of 300 000 PBMCs in 200 μL/well of a 96‐well plate, in Iscove's Modified Dulbecco's Medium (IMDM; Thermofisher, Waltham, MA, USA) containing 10% human serum (kind gift, Dr. Wayne Dyer, Australian Red Cross Lifeblood, Sydney, Australia), which were incubated with antigen for 44–48 h, in a 5% CO_2_ humidified incubator [9]. Separate cultures were incubated with individual SARS‐CoV‐2 pooled peptide antigens (1 μg/mL, Genscript) as indicated. All experiments additionally included: (i) a culture medium only, negative control well; (ii) anti‐CD3/CD28/CD2 T cell activator—polyclonal positive control well (1:200 dilution, STEMCELL Technologies); and (iii) influenza virus—antigen positive control well (1:200 dilution, Viatris). PBMCs were stained as per flow cytometry details below. Antigen‐specific CD4^+^ T cells were gated as the CD25^+^OX40^+^ % of CD3^+^CD4^+^ T cells [9].

### 4.4 IL‐10 mAb Staining

We conducted IL‐10 staining using intracellular and cytokine capture protocols. For intracellular staining, we expanded PBMCs from a CMV^+^ donor for 8 days with 2 μg/mL of Cytomegalovirus (CMV) grade III antigen (Meridian Life Sciences, Memphis, USA), a lysate of purified CMV antigens from the AD169 strain. Cells were then incubated for 6 h with 25 ng/mL Phorbol 12‐myristate 13‐acetate (PMA) and 1 μg/mL ionomycin in the presence of 10 μg/mL Brefeldin A (Invitrogen). Negative controls did not receive PMA, ionomycin, or Brefeldin A. Intracellular IL‐10 staining with a 1:100 dilution of IL‐10‐PECy7 (JES3‐19F1) (BD Biosciences) was done after fixation and permeabilization with FoxP3 transcription factor buffer set as detailed above. For IL‐10 cytokine capture staining, healthy PBMCs were stimulated with 1 μL/mL of CytoStim (Miltenyi Biotec, #130‐092‐172) for 48 h. The surface mAb panel included 1:20 of the IL‐10 capture mAb that is bispecific for the CD45 receptor and IL‐10 (Miltenyi Biotec, #130‐090‐434). After 5 min staining, cells were adjusted to 1 × 10^6^ cells/mL in a 15 mL tube by adding ImmunoCult (STEMCELL Technologies). Cells were then stimulated with 1:200 ImmunoCult Human CD3/CD28/CD2 T Cell Activator (STEMCELL Technologies) while rotating at 37°C for 1 h. PBS was added and cells centrifuged for 5 min at 500 × *g*. Cells were then stained with 1:10 of the IL‐10‐PE detection antibody with fixable viability dye for 15 min followed by intracellular staining for BLIMP‐1‐AF647 (6D3) using the Foxp3 buffer fixation/permeabilization set as described above.

### 4.5 Single‐Cell RNA‐Seq Analysis of Antigen‐Specific T Cells

CD25/OX40 AIM assays were also conducted with PBMC from 4 donors from the ADAPT study were cultured for 48‐h with Wuhan SARS‐CoV‐2 nucleoprotein, spike or spike RBD peptide pools (1 μg/mL, Genscript) and *Staphylococcus* enterotoxin B (SEB; 1 μg/mL; Sigma‐Aldrich). A total of 14 053 SARS‐CoV‐2‐specific CD25^+^OX40^+^ CD4 T cells from these cultures were purified by cell sorting. Unstimulated purified ex vivo memory CD4 T cells (CD3^+^CD4^+^CD45RO^+^) were used as a comparator subset. The mAb panel included: CD3 (Clone: UCHT1), CD4 (RPA‐T4), CD8 (RPA‐T8), CD39 (A1), CD69 (FN50) all BioLegend, CD25 (2A3), OX40 (L106) from BD Biosciences and CD45RO (UCHL1) from Beckman Coulter. Purities of sorted populations were > 99% (Figure S1b,c). Populations were individually stained with Total‐Seq C hashtags (BioLegend), and single cell libraries were generated using the 5′v2 Gene expression and immune profiling kit (10× Genomics). Subsequent cDNA and TCR libraries were generated according to manufacturer's instructions. Generated libraries were sequenced on the NovaSeq S4 flow cell (Illumina) at Read 1 = 28, i7 index = 10, i5 index = 10 and Read 2 = 90 cycles according to manufacturer's instructions.

### 4.6 RT‐qPCR

Real‐time PCR (RT‐qPCR) was performed using either the IQ5 RT‐PCR detection system (Bio‐Rad) or the Light Cycler 480 system (ROCHE). Taqman primers/probes and microRNA detection probes were previously described [16]. Additional probes used include IL‐2 (Hs00174114_m1), IL‐10 (Hs00961622_m1), and FOXP3 (forward primer: TCACCTACGCCACGCTCAT; reverse primer: TCATTGAGTGTGTCCGCTGCTT; probe: TGGCCATCCTGGAGGCTCC). Primers and probes for β‐actin were as described previously [30].

### 4.7 Microarray

After cell sorting, RNA was isolated using TRIzol reagent (Invitrogen, see RNA extraction) and prepared for hybridization to microarrays (900 470 U133 Plus 2.0 arrays; Affymetrix). The array data were normalized by dividing the signal of each gene by the median intensity of the chip. Each gene was divided by the median of its measurements in all samples and processed using Filemaker Pro 5, allowing formation of clusters based on intensity values and fold changes, or functionality (based on ontologies derived from Affymetrix, updated mid 2008). Commonalities/exclusions between gene sets were determined using AND/OR/NOT logic, identifying groups of genes following specific patterns of transcriptional regulation.

### 4.8 Transcriptomic Analysis of Generated Data

#### 4.8.1 Pre‐Processing of Raw Sequencing Files

Single‐cell sequencing data was aligned and quantified using Cell Ranger (10× Genomics) against the human reference genome (10× Genomics, July 7, 2020, release) with default parameters. Raw hashtag data was processed using CITE‐Seq count algorithm. Cell conditions were demultiplexed using “HTODemux” function implemented in Seurat. Filtering and quality control were performed using Seurat (39) on data containing 15 276 cells where 14 053 cells were retained satisfying thresholds of both < 10% mitochondria content and the number of genes between 300 and 5000. “SCTransform” was used for normalization. Genes encoding BCR and TCR were removed to improve cell clustering. Principal Component Analysis (PCA) dimensional reduction was performed on variable genes identified by ‘VariableFeatures’ function and cell clusters were visualized using Uniform Manifold Approximation and Projection (UMAP) clustering implemented in Seurat.

#### 4.8.2 Annotation of Cell Identities and Differential Gene Expression Analysis

T‐cell sub‐populations were manually annotated based on UMAP clustering and markers defined by ‘FindAllMarkers’ function in Seurat. Raw counts from defined cell populations were normalized using scran/scater and differential gene expression analysis was performed using Limma voom.

### 4.9 Transcriptomic Analysis of Public Data

Single cell multi‐omic data were obtained from the Gene Expression Omnibus (GEO) under accession GSE261278. Sample preparation and data processing are described in the original study [20] GEX and CITE‐seq count matrices provided by the authors were downloaded with sample metadata. The metadata provided contained existing cell classification tags (regulatory, naïve, effector or central memory) by MultiModal Classifier Hierarchy (MMoCHi). Downstream, data were analyzed in R Studio (4.5.0) using the R packages Seurat, edgeR, limma, and ggplot2. The analysis was restricted to CD4^+^ T cells derived from lung and lung‐draining lymph nodes sorted as SARS‐CoV‐2 spike‐specific.

#### 4.9.1 Stratification of Tregs by PRDM1 Expression

To investigate transcriptional differences associated with BLIMP‐1 expression, Tregs were stratified based on RNA expression of the gene *PRDM1*. Cells were classified into two groups based on *PRDM1* expression thresholds (PRDM1 low expression < 0.32 and PRDM1 high expression > 0.70) for downstream differential gene expression analysis (Figure S2c).

#### 4.9.2 Pseudobulk Aggregation and Differential Gene Expression Analysis

To reduce single‐cell sampling noise and appropriately model donor‐level replication, raw counts were summed across cells to generate a pseudobulk expression profile. Pseudobulked samples containing fewer than 10 cells were excluded. The pseudobulk matrix generated for RNA analysis comparing spike‐specific Tregs and non‐Tregs is attached as a Data File S1. Differential expression between PRDM1‐high and PRDM1‐low cells used a pseudobulk workflow based on the limma‐voom framework. Raw count matrices were processed with the edgeR DGEList pipeline, followed by filtering of lowly expressed genes using filterByExpr. Library sizes were normalized using the trimmed mean of M‐values (TMM) method. Mean–variance relationships were modeled using the voom transformation, and linear models were fitted using the limma package with empirical Bayes moderation. The design matrix included the covariates of donor identity, tissue of origin, stimulation condition, and *PRDM1* expression group where applicable. Differentially expressed genes were ranked by moderated t‐statistics and adjusted for multiple testing using the Benjamini–Hochberg false discovery rate (FDR).

### 4.10 Statistics

Wilcoxon paired *t‐*tests were used to analyze paired samples. For unpaired samples, the Mann–Whitney U test was used. All statistical analysis was done using the Prism v9 software (GraphPad, La Jolla, CA, USA); *p* values < 0.05 were considered significant.

## Author Contributions

**Chansavath Phetsouphanh:** writing – original draft, methodology, validation, writing – review and editing, formal analysis, investigation, visualization, data curation. **Katherine J. L. Jackson:** writing – review and editing, formal analysis, methodology. **Vera Klemm:** methodology, writing – review and editing, formal analysis. **John J. Zaunders:** conceptualization, writing – review and editing, writing – original draft, formal analysis, supervision, methodology, validation, investigation, funding acquisition, visualization, data curation. **Nabila Seddiki:** conceptualization, investigation, writing – original draft, methodology, validation, visualization, writing – review and editing, formal analysis, project administration, supervision, data curation, funding acquisition. **Anthony D. Kelleher:** conceptualization, funding acquisition, writing – review and editing, validation, supervision, resources, methodology, project administration, visualization. **Denise C. Hsu:** methodology, writing – review and editing, formal analysis. **Yin Xu:** methodology, writing – review and editing, formal analysis. **Bikash Manandhar:** methodology, formal analysis, writing – review and editing. **Deirdre Cuff:** formal analysis. **Laura Cook:** investigation, writing – original draft, methodology, validation, writing – review and editing, formal analysis, visualization, data curation.

## Funding

This work was supported by the National Health and Medical Research Council (510448). St Vincent’s Hospital Foundation grant (MRF2001684).

## Disclosure

N.S., J.J.Z. and A.D.K. hold patent applications for the CD25^+^CD134^+^CD39^+^ (PCT/AU2008/001407, 2008) and the CD25^+^CD127^low^ assays (PCT/AU2006/001080, 2007).

J.J.Z. and A.D.K. hold a patent application for the CD25^+^CD134^+^ assay (WO/2007/106939).

## Conflicts of Interest

The authors declare no conflicts of interest.

## Supporting information


**Data S1:** imcb70148‐sup‐0001‐DataS1.xlsx.

**Figure S1:** Gene annotation and sorting strategy.
**Figure S2:** BLIMP‐1 and IL‐10 staining and PRDM1 expression cutoffs.

## Data Availability

Single‐cell RNA‐seq datasets presented in this study can be found online in the Sequence read Archive (SRA) database; accession number PRJNA877691 ID: 877691. We re‐analyzed data from the Gene Expression Omnibus (GEO) under accession GSE261278. Microarray data can be found using the accession number (GSE336394).
