# Supplementary material for: BLIMP‐1 and CD39 expression define human effector memory regulatory T cells in blood and recall responses to viruses
Source: Immunol Cell Biol. 2026 Jul 14;104(8):757–67. doi: 10.1111/imcb.70148 (PMC13542746; doi:10.1111/imcb.70148)
Supplement: Supplementary file 1 [file IMCB-104-757-s002.pdf]

Supplemental Data

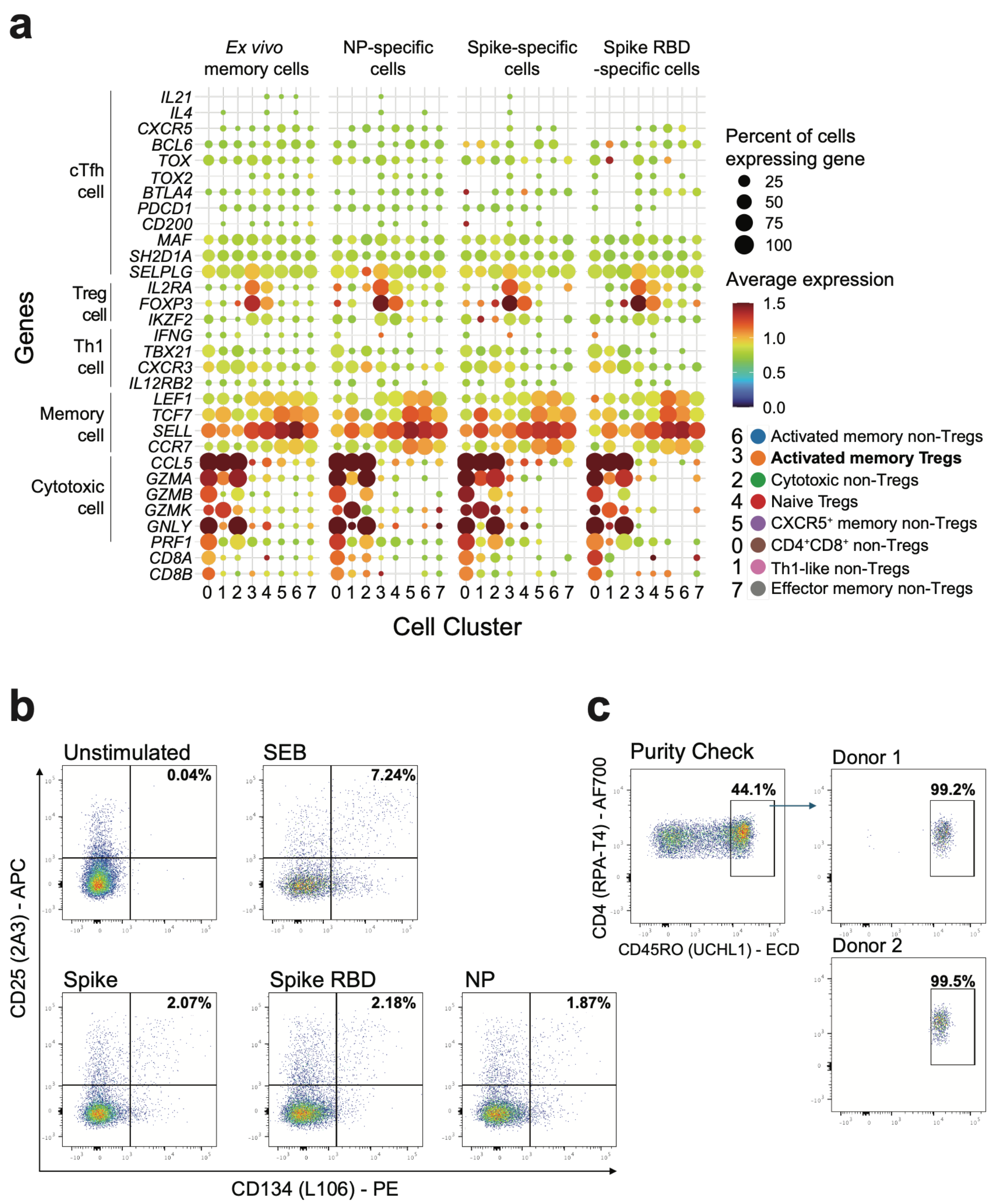

Supplemental Figure 1. Gene annotation and sorting strategy.

(**a**) Dot plot showing average expression of T cell lineage-defining genes and the percentage of cellular expression within each cluster. (**b**) Representative plot gated on live CD4$^+$ T cells following 48-hour stimulation with the specified SARS-CoV-2 peptide pools, with SEB as a positive control. Antigen-specific CD4$^+$ T cells were bulk sorted from the CD25$^+$CD134$^+$ quadrant on the BD Aria III. **(c)** Memory CD45RO$^+$ *ex vivo* (unstimulated) CD4$^+$ T cells were bulk sorted for 10X sequencing. A purity of >99% was achieved for all samples.

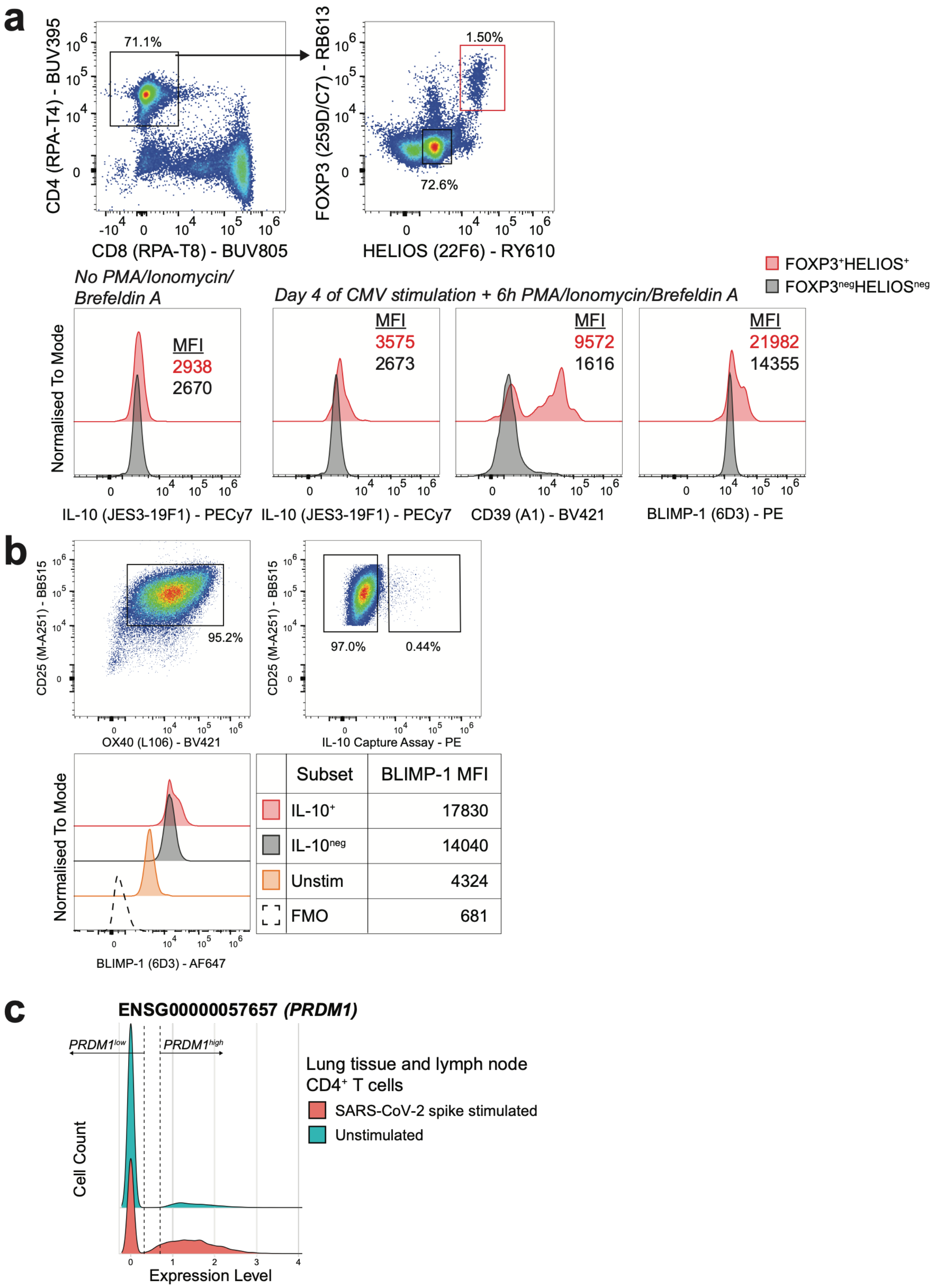

Supplemental Figure 2. BLIMP-1 and IL-10 staining and *PRDM1* expression cutoffs.

(a) CMV seropositive donor PBMCs were expanded for 8-days with CMV lysate then stimulated with PMA and Ionomycin in presence of Brefeldin A for 6 hours for intracellular cytokine staining Cells were gated as live $CD3^{+}CD4^{+}CD8^{neg}$ then either $FOXP3^{+}HELIOS^{+}$ Tregs (red) or $FOXP3^{neg}HELIOS^{neg}$ non-Tregs (black). Expression of IL-10, CD39 and BLIMP-1 is shown along with no PMA/Ionomycin/Brefeldin A control for IL-10. Data are

from a single experiment. **(b)** Healthy donor PBMCs were stimulated with CytoStim™ for 48 hours, surface stained for mAbs including IL-10 capture mAb, stimulated with anti-CD3/CD28/CD2 for 1 hour then stained with IL-10-PE detection antibody. The $CD3^+CD4^+CD25^+OX40^+$ T cells were divided into IL-10^+ and IL-10^{neg} and levels of BLIMP-1 shown, along with unstimulated and FMO controls. Data are from a single experiment. **(c)** Analysis of scRNA-Seq data from Davis-Porada *et al.,*[1] for lung tissue and lung lymph node $CD4^+$ T cells that were stimulated with SARS-CoV-2 spike (3,954 cells) or were unstimulated (31,814 cells). Dotted lines show the expression level cutoffs used to define *PRDM1*^{low} and *PRDM1*^{high} cells for analysis in Figure 4.

Reference

1. Davis-Porada, J., *et al.* Maintenance and functional regulation of immune memory to COVID-19 vaccines in tissues. *Immunity* **57**, 2895-2913 e2898 (2024).
